# LB1576. High Prevalence of Oral *Treponema pallidum* by PCR among US Adults with Early Syphilis

**DOI:** 10.1093/ofid/ofac492.1886

**Published:** 2022-12-15

**Authors:** Jodie Dionne, Lorenzo Giacani, Emily Romeis, Alexander Boutwell, Lori Newman, Kimberly Workowski, Edward Hook

**Affiliations:** University of Alabama at Birmingham, Birmingham, Alabama; University of Washington, Seattle, Washington; University of Washington, Seattle, Washington; University of Alabama at Birmingham, Birmingham, Alabama; NIH/NIAID, Rockville, Maryland; Emory University, Atlanta, Georgia; University of Alabama at Birmingham, Birmingham, Alabama

## Abstract

**Background:**

*Treponema pallidum* (*T. pallidum*) is transmitted from person to person by direct contact with widespread dissemination early after acquisition. Current syphilis diagnostic testing is limited to darkfield microscopy from visible lesions and serologic antibody detection. *T. pallidum* molecular diagnostics are urgently needed.

**Methods:**

As part of a multicenter US syphilis treatment trial that completed enrollment in March 2022 (NCT 3637660), we randomized 249 adults with early syphilis infection to 1 vs 3 weekly doses of benzathine penicillin G to compare treatment response at 6 months. In a small substudy (n=32) presented here, consenting participants at the University of Alabama at Birmingham (UAB) and Emory University had pre-treatment oral (buccal mucosa and posterior pharynx) and lesion swab samples collected. Following DNA extraction, *T. pallidum* burden in samples was quantified by targeting the *tp0574* gene. Syphilis was staged by experienced providers according to physical examination and serology.

**Results:**

Study participants were men (100%) and most were living with HIV (91%) with mean CD4 count 477 cells/mm^3^. Syphilis stage was categorized as primary in 6 (19%), secondary in 18 (56%) and early latent in 8 (25%). Oral swab qPCR positivity rates were 17% in primary disease, 44% in secondary disease, and 63% in early latent disease (see Figure). Lesion PCR positivity was similar in primary and secondary syphilis (40% and 38.5%). Mean treponemal burden was highest on oral swabs of participants with early latent syphilis (13,128,969) and lesion swabs of participants with secondary syphilis (2,033,309).

**Figure:**
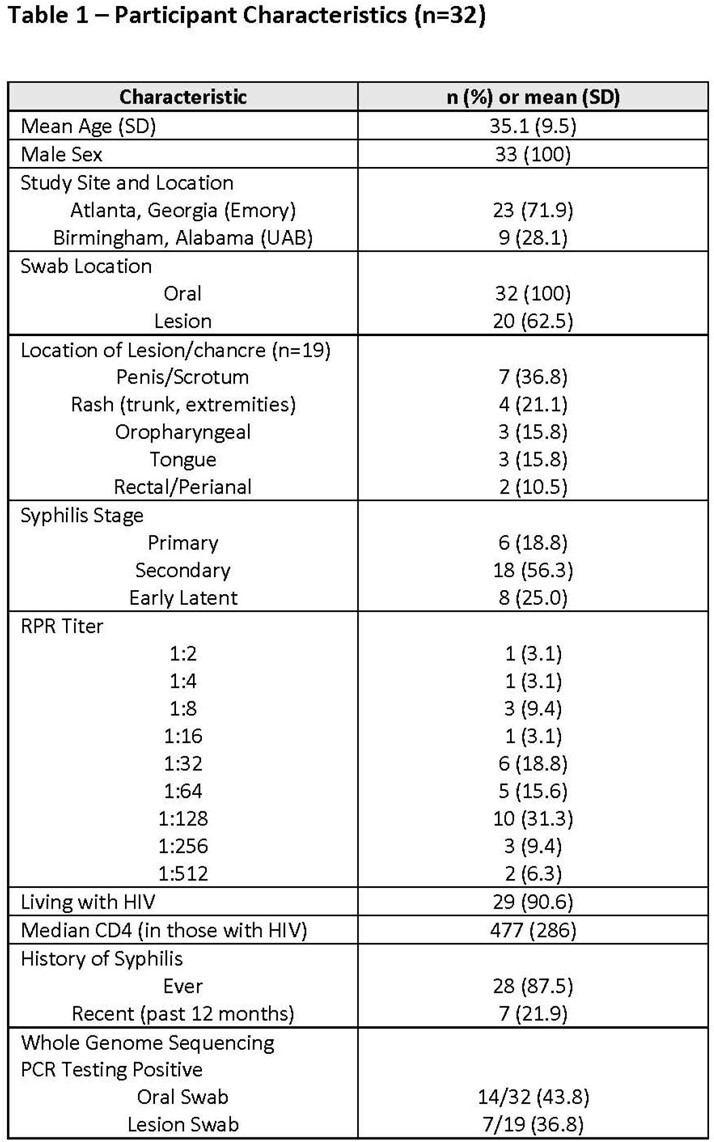

**Figure:**
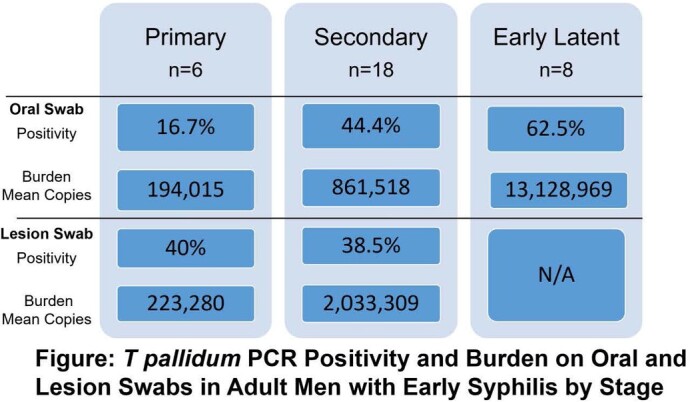

**Conclusion:**

Among adult males, many living with HIV, data suggest a role for oral and lesion swabs for the molecular detection of *T. pallidum.* Widespread availability of PCR, the relative ease of oral specimen collection, and the opportunity to directly demonstrate *T. pallidum* in persons with early latent syphilis suggest the opportunity for PCR testing to enhance and refine syphilis diagnostic testing. The elevated proportion of positive oral samples and significant organism burden in secondary and early latent syphilis is consistent with potential for transmission.

**Disclosures:**

**All Authors**: No reported disclosures.

